# Integrated Primary Care Model Reduces High‐Risk Medications for People Living with Dementia

**DOI:** 10.1002/alz.086128

**Published:** 2025-01-09

**Authors:** Carolyn K Clevenger, Anjali Khakharia, Laura J Medders, Miranda Moore

**Affiliations:** ^1^ Emory University, Atlanta, GA USA; ^2^ Emory Healthcare, Atlanta, GA USA

## Abstract

**Background:**

Harmful care including the prescribing of high‐risk and potentially inappropriate medications for older people is widespread among older adults, including people living with dementia (PLWD). Integrated Memory Care (IMC) is a comprehensive dementia care model where patients and their family caregivers access dementia‐sensitive geriatric primary care.

**Methods:**

We conducted a retrospective observational study of adult patients of IMC, Cognitive Neurology (CN), and Primary Care (PC) clinics aged 65 and older with a diagnosis of dementia in 2019‐2021. We matched patients by age, gender and race and measured the hospitalization, rate of deprescribing and inappropriate screening test referrals using logistic regressions controlling for clinic. Additionally, we conducted a regression adjusted for state of illness (proxied by activities of daily living (ADL)) for the IMC and CN clinics.

**Results:**

Overall, 509 patients seen in IMC were matched with 490 CN patients and 509 PC patients. Most patients were female and white and aged 70‐85. IMC patients had higher overall ADL scores than CN indicating more functional dependence.

IMC patients had higher odds of deprescribing high dose Antipsychotics (OR: 4.383, CI:1.405,13.677), benzodiazepines (OR: 3.338, CI: 1.541,7.231) and Opiates (OR: 1.004, CI: 0.415, 2.431) when compared to CN patients. After adjusting for ADL scores, the odds ratios were 4.952 (1.509,16.25), 3.434 (1.573, 7.494) and 0.991 (0.408, 2.404) respectively.

IMC also had higher odds of deprescribing high dose Antipsychotics (OR 5.538 CI: 1.21,25.359), benzodiazepines (2.632, CI: 1.297, 5.341) and Opiates (1.424, CI: 0.571, 3.55) when compared to PC patients.

**Conclusions:**

Patients managed in a dementia‐sensitive primary care practice were deprescribed high‐risk medications after one‐year of management, reducing avoidable adverse events.

